# Mid-Term Changes in Quality of Life and Nutritional Habits Following Gastric Bypass: A 24-Month Follow-Up Study

**DOI:** 10.3390/nu18020288

**Published:** 2026-01-16

**Authors:** María Antonia Martínez-Sánchez, Inmaculada Ros-Madrid, Virginia Esperanza Fernández-Ruiz, Rosario Paloma Cano-Mármol, Juan José Hernández-Morante, María Ángeles Núñez-Sánchez, Andrés Balaguer-Román, María Dolores Frutos-Bernal, Antonio José Ruiz-Alcaraz, María Isabel Queipo-Ortuño, Mercedes Ferrer-Gómez, Bruno Ramos-Molina

**Affiliations:** 1 Obesity, Diabetes and Metabolism Laboratory, Biomedical Research Institute of Murcia (IMIB), 30120 Murcia, Spain; mariaantonia.martinez1@gmail.com (M.A.M.-S.); inmarosmadrid@gmail.com (I.R.-M.); virginiaesperanza.fernandez@um.es (V.E.F.-R.); rosariopaloma.cano@um.es (R.P.C.-M.); jjhernandez@ucam.edu (J.J.H.-M.); mangelesnunezsanchez@gmail.com (M.Á.N.-S.); a.balaguerroman@gmail.com (A.B.-R.); 2 Department of Endocrinology and Nutrition, Virgen de la Arrixaca University Hospital, 30120 Murcia, Spain; 3 Eating Disorders Research Unit, Faculty of Nursing, UCAM Catholic University of Murcia, 30107 Murcia, Spain; 4 Department of General and Digestive System Surgery, Virgen de la Arrixaca University Hospital, 30120 Murcia, Spain; mfb25s@gmail.com; 5 Department of Biochemistry, Molecular Biology B and Immunology, Faculty of Medicine, University of Murcia, 30120 Murcia, Spain; ajruiz@um.es; 6 Intercenter Medical Oncology Clinical Management Unit, Regional and Virgen de la Victoria University Hospitals, Málaga Biomedical Research Institute (IBIMA)-CIMES-UMA, 29010 Málaga, Spain; maribelqo@gmail.com; 7 Department of Surgical Specialties, Biochemical and Immunology, Faculty of Medicine, University of Málaga, 29071 Malaga, Spain

**Keywords:** gastric bypass, obesity, nutritional habits, quality of life, mental health

## Abstract

**Background/Objectives:** Obesity is an increasingly concerning public health issue due to its high prevalence and its association with multiple comorbidities. A significant proportion of patients with obesity who undergo bariatric surgery could exhibit suboptimal mid-term outcomes. This study aims to comprehensively assess anthropometric, clinical, biochemical, nutritional, and quality of life parameters in patients with severe obesity undergoing bariatric surgery, with a particular focus on outcomes at 24 months post-surgery to capture mid-term effects that may not be apparent during the first year of follow-up. **Methods:** A prospective study was conducted in 95 patients with obesity undergoing bariatric surgery (Roux-en Y gastric bypass; RYGB) at the Virgen de la Arrixaca University Clinical Hospital (Murcia, Spain) between 2020 and 2023. Participants were followed up at 6, 12, and 24 months after RYGB. The study incorporated anthropometric assessments (BMI, body composition via bioelectrical impedance), full biochemical profiling, dietary analysis (using a validated food frequency questionnaire), and quality of life assessment (SF-36 questionnaire). **Results:** Our results showed significant weight loss after the intervention, accompanied by improvements in metabolic parameters, and dietary habits. Regarding quality of life, significant improvements were observed in both the physical (baseline: 39.62%; 6 months: 52.40%; 12 months: 53.12%) and mental components (baseline: 42.08; 6 months: 53.40; 12 months: 52.14%) at 6 and 12 months post-surgery. However, our prospective 24-month follow-up revealed that, despite these initial benefits, mental health significantly declined compared with the 12-month follow-up (24 months: 46.85%). In contrast, the physical component remained relatively stable at 24 months (24 months: 50.91%). However, our prospective 24-month follow-up revealed that, despite these initial benefits, there was a decline in mental health compared to the 12-month follow-up. **Conclusions:** While bariatric surgery is associated with improvements in anthropometric measures and some aspects of quality of life, our findings underscore the need for continued mid-term support to address emerging challenges in mental well-being.

## 1. Introduction

Severe obesity (BMI ≥ 35 kg/m^2^) has become a global public health problem, increasing the risk of developing chronic diseases such as type 2 diabetes, cardiovascular disease (CVD), chronic liver diseases, and certain types of cancer [1]. In 2020, approximately 4.9% of the adult Spanish population was classified as severely obese [2]. Despite the health issues faced by patients with severe obesity and the economic costs associated with this condition, the underlying factors contributing to the prevalence of severe obesity are still not fully understood.

The treatment of severe obesity is complex and requires a multidisciplinary approach that addresses both the medical and psychosocial aspects of the patients. While lifestyle changes, such as a healthy diet and increased physical activity, are fundamental, many patients with severe obesity fail to maintain significant mid-term weight loss through these methods. Consequently, bariatric surgery has emerged as an effective therapeutic option for achieving sustained weight reduction and improving obesity-related comorbidities [3,4].

However, despite the high effectiveness of bariatric surgery in many patients with severe obesity, not all achieve successful mid-term outcomes. It is estimated that approximately 20% to 30% of patients undergoing this type of intervention experience what is considered a “treatment failure.” This failure can manifest in various ways, such as suboptimal clinical response (TWL < 20%, absence of improvement or clinical worsening) or recurrent weight gain (exceeding 30% of the TWL, as well as recurrence or deterioration of obesity-related comorbidities) [5,6]. Among the main causes of failure is poor adherence to lifestyle changes, as many patients fail to maintain post-surgical recommendations related to diet and physical activity, which can lead to the recovery of lost weight [7,8,9].

One of the principal goals of surgical treatment for obesity, beyond reducing mortality and morbidity, is to achieve lasting improvement in quality of life [10]. Quality of life has been recognized as an important health indicator for both the general population and for individuals suffering from chronic or potentially life-threatening diseases [11]. Patients increasingly seek surgical care due to deterioration in their quality of life, and indeed, improvement in this area is often considered an indicator of the effectiveness of the treatment [12].

On the other hand, monitoring nutritional habits in bariatric patients is essential during clinical follow-up, as surgery alters digestive anatomy and requires sustained dietary changes to ensure mid-term success. Moreover, Roux-en-Y gastric bypass (RYGB) often leads to nutritional deficiencies that require lifelong vitamin and mineral supplementation. The most frequently observed deficiencies include vitamin B12, iron, calcium, vitamin D, and folic acid, which, if not properly corrected, may result in anemia, bone demineralization, and neurological symptoms [13]. Follow-up assessments are typically recommended at 1, 3, 6, and 12 months postoperatively [14]. One notable effect of bariatric surgery is the change in body composition, characterized by a reduction in both fat and lean mass compartments at 12 months after the intervention, which is greater than that observed in patients treated with diet and exercise alone [15]. Therefore, to help prevent excessive lean mass loss, it is recommended to ensure a minimum protein intake of 60 g/day, up to 1–1.5 g/kg/day, combined with regular physical activity [14]. Inadequate intake can lead to nutritional deficiencies, loss of lean mass, or weight regain. Longitudinal dietary assessments enable early identification of deviations, allow for timely nutritional support, and promote sustained adherence to postoperative recommendations, ultimately supporting optimal metabolic and functional recovery [16].

The main objective of this study is to comprehensively analyze patients with severe obesity undergoing bariatric surgery, with a particular focus on evaluating the mid-term impact of the intervention, especially at 24 months post-surgery, on anthropometric, clinical, biochemical, nutritional, and quality of life parameters.

## 2. Materials and Methods

### 2.1. Study Design and Subjects

This study included patients with severe obesity recruited to undergo RYGB at the Virgen de la Arrixaca University Clinical Hospital (HCUVA, Murcia, Spain) between January 2020 and December 2023. Inclusion criteria were: men and women with a BMI ≥ 35 kg/m^2^, a duration of obesity of at least 5 years, and ages between 18 and 65 years. All patients were followed by a dietitian before surgery, with scheduled visits every two months, for a minimum of four visits prior to the procedure. After surgery, patients were evaluated by a dietitian before hospital discharge, again at one week postoperatively, and subsequently every 15 days until they were able to tolerate a solid diet. Thereafter, monthly visits were performed up to 6 months, followed by bimonthly visits until 24 months after surgery.

The postoperative dietary protocol consisted of the following sequential phases: a liquid diet for 1 week, a semi-liquid diet for 2 weeks, a puréed diet for 2 weeks, a soft-chew diet for 2 weeks, and a solid diet for 2 weeks, after which patients were transitioned to a standard Mediterranean diet. From this point onward, patients were not given social or cultural restrictions regarding food choices. Instead, they were advised to adhere consistently to the recommended diet and to limit deviations to no more than once per week. Patients received systematic multivitamin supplementation, and additional vitamins and minerals were supplemented according to identified deficiencies and general clinical guidelines [14].

The study was conducted in accordance with the Declaration of Helsinki and complied with local and national regulations. It was approved by the Clinical Research Ethics Committee of HCUVA (reference number 2020-2-4-HCUVA). Written informed consent was obtained from each participant prior to inclusion in the study.

### 2.2. Anthropometric Measurements

Body weight was measured using a calibrated digital scale with a precision of ±0.1 kg, with participants wearing light clothing and barefoot. Height was recorded in an upright position, without shoes, with a precision of ±0.5 cm. BMI was calculated using the Quetelet formula:

BMI (kg/m^2^) = *weight* (kg)/[*height* (m)]^2^

Waist circumference was measured in an upright position at the horizontal plane above the iliac crest using a measuring tape with a precision of ±0.5 cm. Visceral obesity was defined as waist circumference >80 cm in women and >94 cm in men. Blood pressure was measured twice using an aneroid sphygmomanometer while the patient was seated after 15 min of rest. The average of the two measurements was recorded.

Body composition was assessed using a 50 kHz bioelectrical impedance analyzer (Tanita MC-980MA-N plus, Tanita Inc. Tokio (Japan)), employing the measurement technique between both legs, following the manufacturer’s instructions and predictive formulas embedded in the device. According to the device’s initial calibration, the margin of error is ±2% for impedance measurement and ±0.2 kg for body weight.

Changes in BMI after bariatric surgery were calculated by comparing the initial BMI with the BMI at 12 and 24 months post-surgery (initial BMI−postoperative BMI). Total weight loss (TWL) was calculated as:

TWL (kg) = *Initial weight* − *Postoperative weight*

The percentage of total weight loss (%TWL) was calculated as:

%TWL = (*Initial weight* − *Postoperative weight*)/*Initial weight* × 100

The percentage of excess weight loss (%EWL) was calculated as:

%EWL = (*Initial weight* − *Postoperative weight*)/(*Initial weight* − *Ideal body weight*) × 100

The Broca Index (BI) was used to calculate the ideal body weight (IBW):

BI = *Height in centimeters* − 100 = *IBW*

### 2.3. Functional Evaluation

Functional evaluation was assessed using the Six-Minute Walk Test (6MWT), a submaximal exercise test that evaluates aerobic capacity and the overall physiological response to physical effort. The test involved measuring the total distance that an individual could walk at a fast but tolerable pace over a six-minute period along a straight 30 m corridor. Patients were allowed to stop and resume walking if needed. During the test, the total distance walked was recorded along with heart rate and oxygen saturation at the end of the test [17].

### 2.4. Biochemical Evaluation

Preoperative clinical data were obtained on the day of surgery after an overnight fast of at least 12 h. Serum was separated by centrifugation for laboratory measurements. Glucose, total cholesterol, high-density lipoprotein cholesterol (HDLc), triglycerides (TG), alanine aminotransferase (ALT), aspartate aminotransferase (AST), gamma-glutamyl transferase (GGT), alkaline phosphatase, ferritin, transferrin, albumin, total bilirubin, total proteins, iron, urea, uric acid, creatinine (Jaffe method), apolipoprotein A-I (Apo-AI), apolipoprotein B (Apo-B), and high-sensitivity *C*-reactive protein were measured using the Cobas c702 analyzer (Roche) following standardized methods. Glycated hemoglobin (HbA1c) levels were measured in blood using the HLC-723G8 analyzer (Tosoh Bioscience). Insulin, *C*-peptide, ferritin, transferrin, folate, and vitamin B12 concentrations were determined using the Cobas e801 analyzer (Roche). Low-density lipoprotein cholesterol (LDLc) levels were calculated using the Friedewald formula:

*LDLc* (mg/dL) = *Total cholesterol* − *HDLc* − (*TG*/5)

Insulin resistance was assessed using the Homeostasis Model Assessment for Insulin Resistance (HOMA-IR), calculated as:

*HOMA-IR* = *Insulin* (μU/mL) × *Glucose* (mmol/L)/22.5

### 2.5. Dietary Habit Recording via Food Frequency Questionnaires

Dietary habits of the patients were recorded before surgery using a previously validated semi-quantitative food frequency questionnaire (FFQ) for the Spanish population [18]. This questionnaire included 146 food items and 9 frequency categories ranging from “never or rarely” to “more than 6 times per day.” The recorded intakes were representative of the participants’ usual diets.

The semi-quantitative FFQ used in this study enabled the recording of a wide variety of fundamental macronutrients and micronutrients to understand the habitual diet of the study subjects. Macronutrients assessed included carbohydrates, differentiated between simple and complex types, with particular emphasis on refined sugars; proteins, specifying their origin (animal or plant-based); and fats, focusing on the intake of saturated, monounsaturated, and polyunsaturated fatty acids, distinguishing healthy fat sources (such as olive oil, avocado, and nuts) from trans fats present in processed foods. Micronutrients evaluated included vitamins and minerals (calcium, magnesium, potassium, iron, and zinc), as well as both soluble and insoluble fiber. Nutrient intake calculations were based on Spanish food composition tables [19], providing precise and population-adapted estimates, thereby offering a comprehensive view of patients’ dietary patterns and their relation to metabolic health conditions.

### 2.6. Quality of Life Assessment Using the SF-36 Questionnaire

Quality of life was assessed using the SF-36 questionnaire, a previously validated tool that measures key health aspects in bariatric patients [20]. The questionnaire was administered either as self-completed by the participants or via a guided interview, depending on the characteristics and needs of each patient, thus ensuring comprehension and ease of response. The average time to complete the questionnaire was 10 to 15 min.

The SF-36 evaluates eight fundamental quality of life domains: physical functioning, role limitations due to physical problems, bodily pain, general health perception, vitality, social functioning, role limitations due to emotional problems, and mental health. Additionally, two summary components were generated: the Physical Component Summary (PCS) and the Mental Component Summary (MCS), which provide an overall view of the individual’s physical and mental health status.

Participant responses were transformed into a standardized scale ranging from 0 to 100, where 0 represents the worst health state and 100 the best. This standardization process allowed consistent interpretation of the results and facilitated comparative analysis among domains, as well as with normative reference data, ensuring the validity and reliability of the quality of life analysis.

### 2.7. Statistical Analysis

Associations between qualitative variables were evaluated using the Chi-square test. For quantitative variables, Pearson or Spearman correlation tests were used depending on the nature and distribution of the data. Differences between groups were assessed using Student’s *t*-test, Mann–Whitney test, or analysis of variance (ANOVA), as appropriate. In the longitudinal analysis, the Wilcoxon signed-rank test or the paired Student’s *t*-test was employed. For comparisons involving two or more groups or treatments, ANOVA was used for independent data and the Friedman test for paired data. All statistical analyses were performed using SPSS software, Armonk, NY, USA (version 20.0).

## 3. Results

More than 200 patients were initially recruited for the study. Ultimately, 95 completed the follow-up assessments at 6, 12, and 24 months after surgery. Table 1 shows the evolution of anthropometric variables, body composition, and blood pressure in patients undergoing RYGB, assessed preoperatively and at 6, 12, and 24 months post-intervention. In general, a significant and sustained reduction was observed in body weight, BMI, waist and hip circumference, body fat percentage, as well as systolic and diastolic blood pressure at both 12 and 24 months compared to baseline values. Total weight loss and excess weight loss stabilized between 12 and 24 months, with no significant differences observed. Fat-free mass, muscle mass, and basal metabolic rate also decreased significantly post-surgery, while total body water percentage increased, reflecting changes in body composition. Peripheral measurements, such as arm and calf circumference, decreased significantly and remained reduced over time.

Table 2 presents the evolution of various biochemical parameters assessed preoperatively and at 6, 12, and 24 months post-surgery. Significant decreases in glucose, glycated hemoglobin, insulin, *C*-peptide, and HOMA-IR levels were observed from 6 months post-surgery, with these improvements remaining stable or continuing at 12 and 24 months (*p* < 0.001), indicating sustained improvement in glucose metabolism and insulin sensitivity. Regarding the lipid profile, triglyceride levels showed a significant and sustained reduction (*p* < 0.001), while HDLc progressively and significantly increased from 6 to 24 months (*p* < 0.001). Total cholesterol and LDLc levels did not change significantly post-surgery. Markers of liver function and inflammation showed a significant decrease in GGT and *C*-reactive protein (*p* < 0.001). AST and ALT showed modest changes, with ALT reaching statistical significance at 24 months (*p* = 0.049). Uric acid levels decreased markedly (*p* < 0.001), while urea levels increased at 12 and 24 months. In terms of iron metabolism, ferritin levels progressively decreased, becoming significant at 24 months (*p* < 0.001), whereas transferrin levels significantly increased only at that time point. Serum iron initially decreased and then progressively recovered, with significant differences observed between preoperative values and those at 6 and 12 months. Nutritional parameters revealed a significant increase in folate levels from 6 months onward (*p* < 0.001), and vitamin B12 levels improved significantly at 24 months (*p* = 0.009). Albumin and total protein levels increased significantly throughout follow-up, likely reflecting improved nutritional status after surgery.

**Figure 1:**
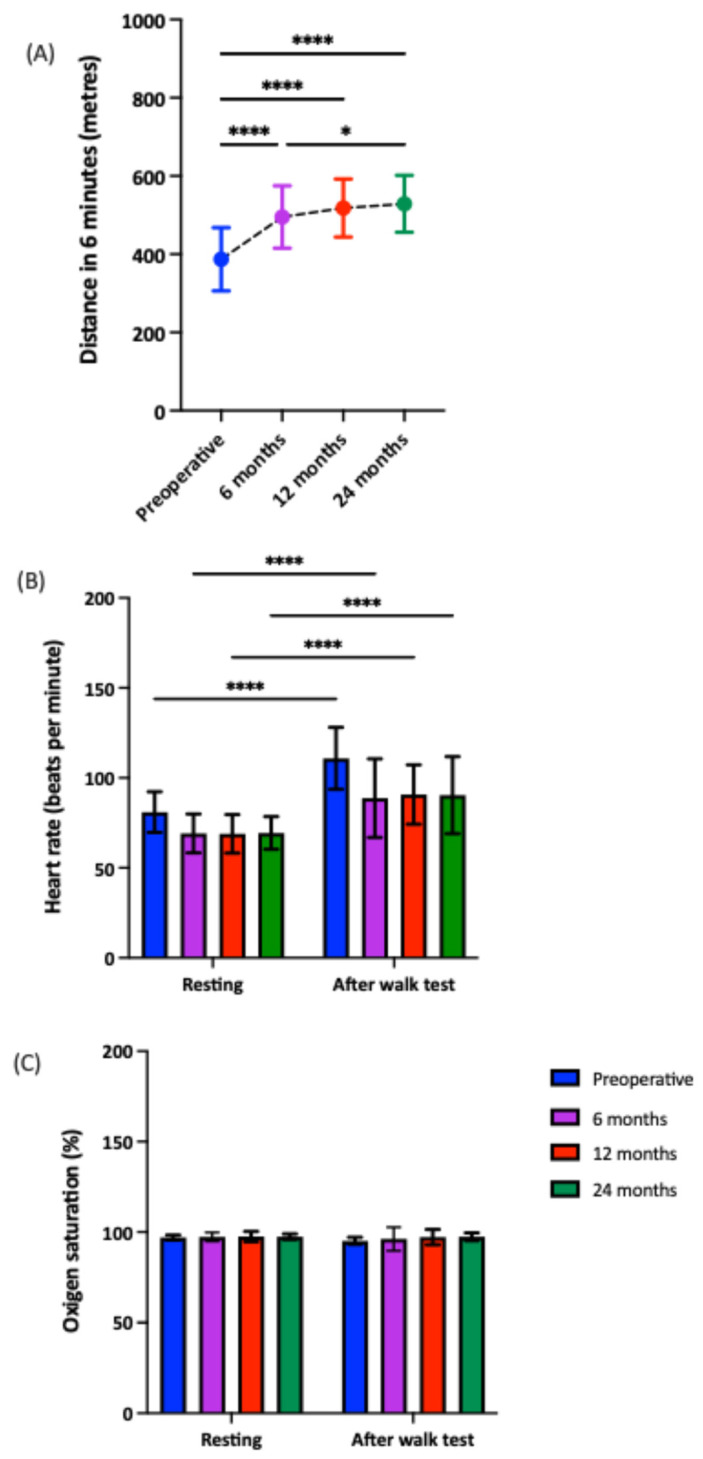
Evolution of functional capacity (distance covered in the 6MWT- (**A**)) and cardiovascular parameters (heart rate (**B**) and oxygen saturation (**C**)) in patients undergoing gastric bypass surgery. Significant differences between groups: * *p* < 0.05; **** *p* < 0.001.

In addition to changes in anthropometric, clinical, and biochemical parameters, we observed notable improvements in functional capacity, as reflected by the distance covered by patients in the 6MWT following surgery (Figure 1). A significant increase was already observed at 6 months postoperatively (*p* < 0.001), which remained stable at the 12- and 24-month follow-up visits (Figure 1A). Regarding cardiovascular variables, RYGB led to a reduction in heart rate, both at rest and after the 6MWT (Figure 1B). However, no significant changes were observed in oxygen saturation levels, either following the 6MWT or after the surgical intervention (Figure 1C).

**Figure 2:**
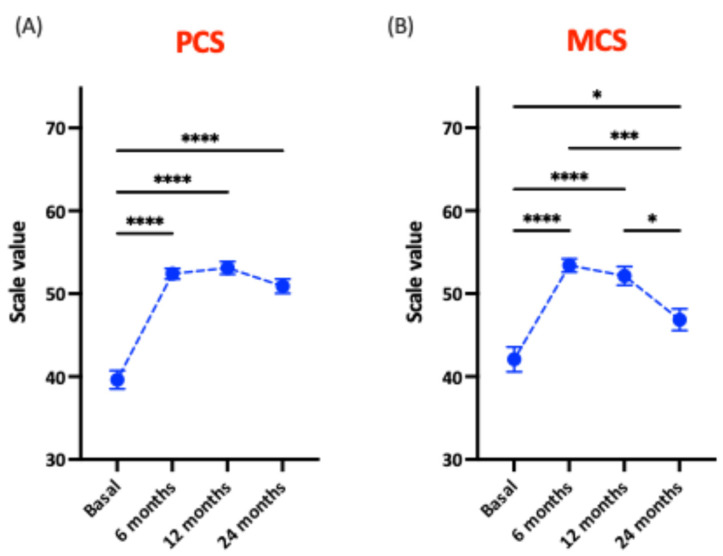
(**A**) Physical (PCS) and (**B**) Mental Component Score (MCS) after bariatric surgery according to the SF-36 questionnaire. Significant differences between groups: * *p* < 0.05; *** *p* < 0.005; **** *p* < 0.001.

Regarding quality of life-related parameters, both the PCS and MCS scores increased significantly at 6 months post-surgery compared to baseline (52.4 and 53.4, respectively) (Figure 2). PCS values at 12 and 24 months (53.1 and 50.9) were similar to those at 6 months, with no significant differences between these postoperative time points (Figure 2A). In contrast, while MCS values at 12 months remained comparable to 6 months, a significant decline was observed at 24 months (Figure 2B).

**Figure 3:**
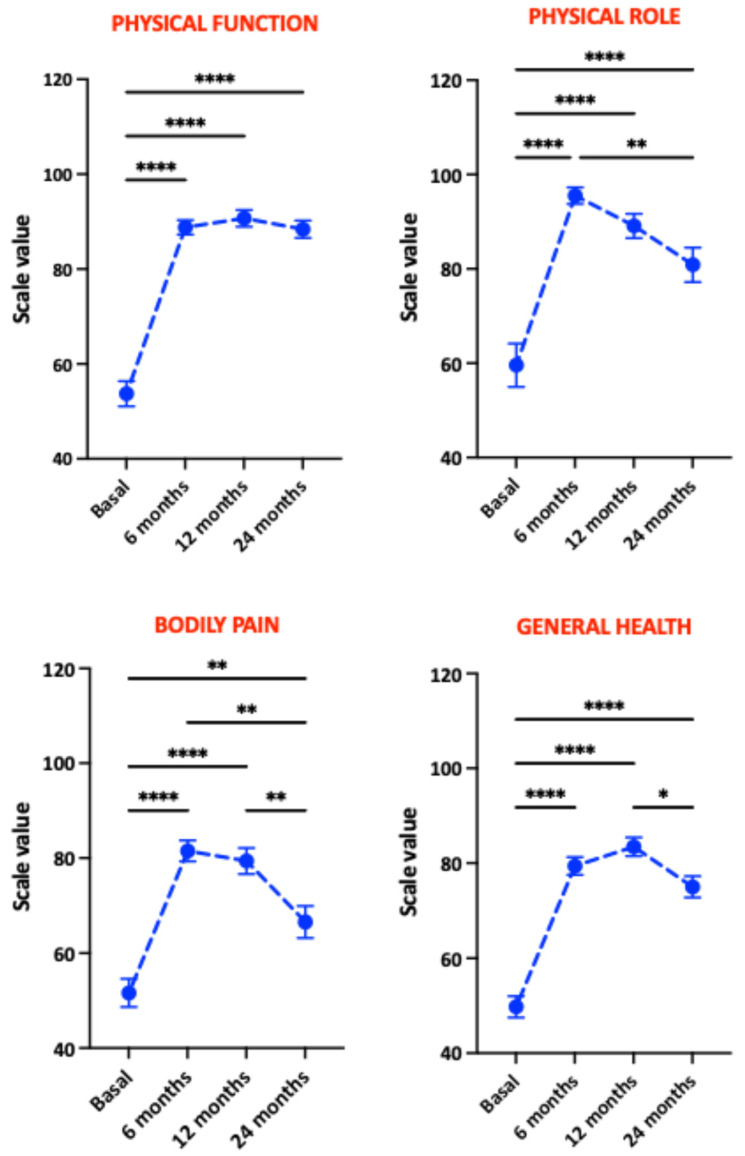
Evolution of the physical health components of the SF-36 questionnaire in patients undergoing bariatric surgery at 6, 12, and 24 months postoperatively. Significant differences between groups: * *p* < 0.05; ** *p* < 0.01; **** *p* < 0.001.

**Figure 4:**
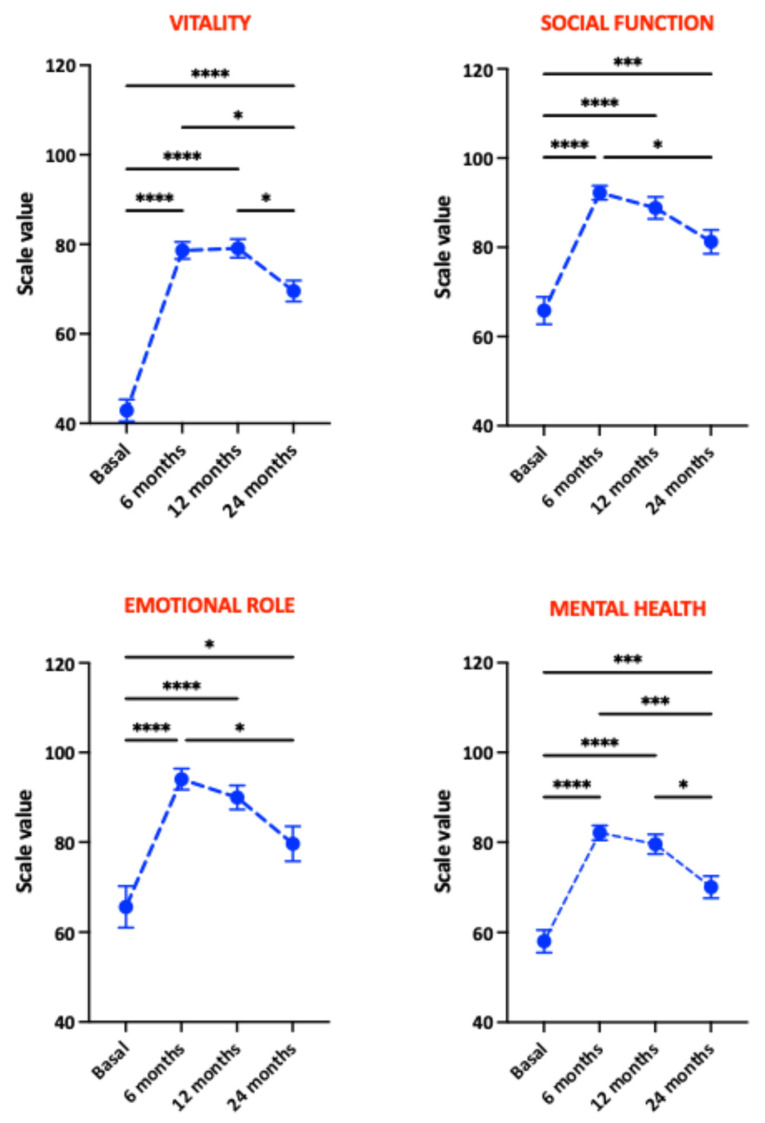
Evaluation of the mental health components of the SF-36 questionnaire in patients undergoing bariatric surgery. Significant differences between groups: * *p* < 0.05; *** *p* < 0.005; **** *p* < 0.001.

The evolution of individual physical health components from the SF-36 questionnaire is illustrated in Figure 3. All physical health components improved significantly. Bodily pain and physical role showed greatest improvements at 6 months, while physical functioning and general health peaked at 12 months. Notably, physical role, bodily pain, and general health scores declined significantly at 24 months, with physical functioning being the only component stable across all three postoperative visits. Figure 4 shows the analysis of mental health components from the SF-36 questionnaire. Vitality increased significantly at 6 months post-surgery and remained stable at 12 months but declined at 24 months. Social functioning, emotional role, and mental health components improved most at 6 months, followed by progressive declines at 12 and 24 months, with the emotional role domain showing the most pronounced decline.

In addition, we evaluated the associations between anthropometric, metabolic, and nutritional intake variables in bariatric patients. Age correlated positively with alcohol consumption (r = 0.235, *p* = 0.022). BMI correlated significantly with several saturated fatty acids, including myristic acid (r = 0.208, *p* = 0.043), palmitic acid (r = 0.236, *p* = 0.021), and stearic acid (r = 0.255, *p* = 0.013), as well as total polyunsaturated fatty acids (r = 0.240, *p* = 0.019), carbohydrates (r = 0.330, *p* = 0.001), dietary fiber (r = 0.234, *p* = 0.023), and total kilocalories (r = 0.275, *p* = 0.007). Waist circumference correlated with carbohydrate intake (r = 0.213, *p* = 0.039) and dietary fiber (r = 0.203, *p* = 0.050). The insulin resistance index (HOMA-IR) correlated significantly with saturated fatty acids such as myristic acid (r = 0.402, *p* < 0.001), palmitic acid (r = 0.336, *p* < 0.001), stearic acid (r = 0.347, *p* < 0.001), and total polyunsaturated fatty acids (r = 0.382, *p* < 0.001). It also correlated with oleic acid (monounsaturated fatty acid) (r = 0.297, *p* = 0.003), total monounsaturated fatty acids (r = 0.318, *p* = 0.002), total fat intake (r = 0.345, *p* < 0.001), and total kilocalories (r = 0.316, *p* = 0.002). No significant HOMA-IR correlations were observed with carbohydrates (r = 0.244, *p* = 0.17) or dietary fiber (r = 0.247, *p* = 0.16).

**Figure 5:**
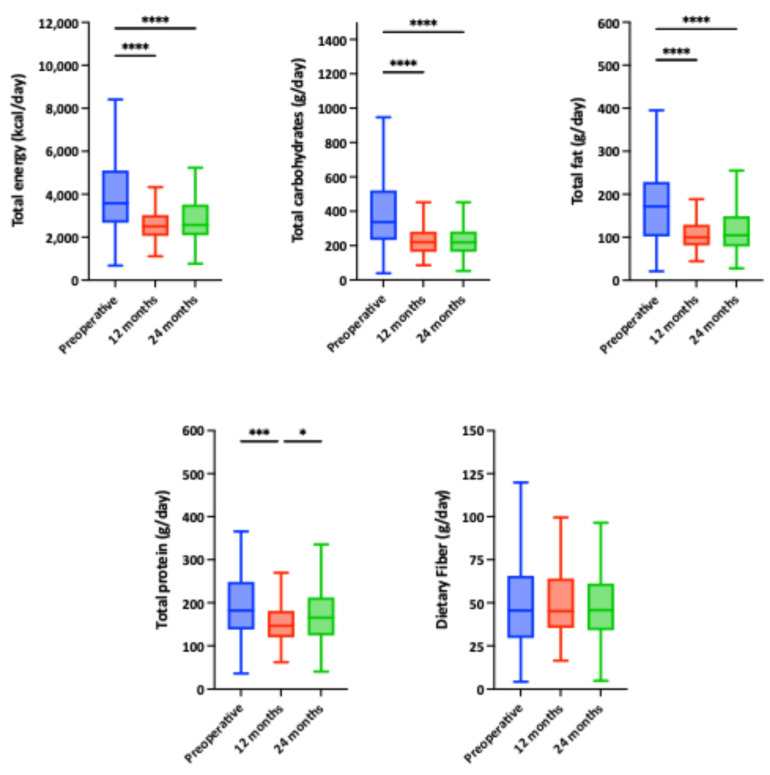
Evolution of macronutrient intake in the diet of patients undergoing bariatric surgery. Significant differences between groups: * *p* < 0.05; *** *p* < 0.005; **** *p* < 0.001.

Figure 5 illustrates the evolution of total energy and macronutrient intake post-surgery. A marked decrease in energy, carbohydrates, fats, and protein intake was observed at one year, remaining stable at 24 months for energy, carbohydrates, and fats, indicating sustained dietary modification. Protein intake showed an increasing trend between 12 and 24 months, possibly reflecting improved dietary adaptation or increased awareness of preserving muscle mass during weight loss. Dietary fiber intake remained stable throughout follow-up. Moreover, Table 3 details alcohol, vitamin, and mineral intake evolution, comparing preoperative values with those at 12 and 24 months. The first postoperative year showed significant reductions in alcohol, cholesterol, B vitamins (riboflavin, thiamine, B6), and minerals such as iron, phosphorus, sodium, zinc, calcium, iodine, and selenium. These likely reflect the impact of the surgery on intake capacity, nutrient absorption, and initial adherence to stricter dietary guidelines. At 24 months, some reductions persisted, but a trend toward partial recovery in the intake of nutrients such as vitamin B12, vitamin D, and certain minerals was observed. Vitamin C intake increased significantly at 12 months and remained stable at 24 months.

Table 4 shows changes in fatty acid intake before and after surgery. A significant reduction in total monounsaturated, polyunsaturated, and saturated fatty acids intake was seen at one year, with stabilization or slight increases at 24 months. Only saturated fatty acids showed a significant increase between 12 and 24 months, suggesting initial dietary quality improvement and global lipid intake reduction, consistent with changes in energy and macronutrient consumption (Figure 5). Among saturated fatty acids, lauric (12:0), myristic (14:0), palmitic (16:0), and stearic (18:0) acids decreased significantly post-surgery, with only myristic acid increasing significantly between 12 and 24 months. Oleic acid (18:1 *n*-9), a monounsaturated fatty acid, decreased significantly at one year, with no significant changes in the second year. Arachidonic acid (20:4 *n*-6) intake decreased slightly at one year but this was not sustained at 24 months. Conversely, long-chain omega-3 fatty acids, eicosapentaenoic acid (EPA, 20:5) and docosahexaenoic acid (DHA, 22:6 *n*-3), increased significantly, indicating qualitative improvements in lipid source choices.

## 4. Discussion

This work addresses two fundamental dimensions for evaluating the success of bariatric surgery, the subjective perception of well-being (quality of life) and changes in nutritional habits. While weight loss and improvement in biochemical parameters are primary objectives in obesity treatment, it is increasingly recognized that psychological well-being and the sustainability of healthy habits are essential components for mid-term therapeutic success.

In this study, a %TWL of 33% was achieved at 12 months, consistent with findings reported by Benaiges et al. [21], and this result was maintained at 24 months. However, the %EWL was 78%, slightly lower; this difference may be explained by the higher BMI of patients in that study compared to our cohort [21]. In this study, patients experienced a mean weight loss of 27.2 kg at 12 months, which is comparable to other studies; however, the FFM loss was 10 kg, which is higher than previously reported [15,22]. Notably, this loss of muscle mass occurred despite a maintained protein intake of over 100 g/day at 24 months post-surgery. However, a sustained improvement in functional capacity and cardiovascular adaptation to exercise was observed following surgery, as reflected in the 6MWT outcomes. Patients showed a 25% increase in functional capacity at one year, consistent with findings by Jassil et al. [23] and de Souza et al. [24], who also reported improvements in cardiovascular performance. However, the study by de Souza et al. [24] reported a higher post-test heart rate than our findings, which could be attributed to the higher BMI of their study participants. Notably, this improvement was maintained at 24 months post-surgery.

Regarding the biochemical profile, improvements were observed in HDLc levels, along with reductions in triglycerides, glucose, and HbA1c, finding consistency with those reported in other studies with follow-up periods extending beyond 24 months [25]. However, unlike our results, other studies have reported significant reductions in LDLc and total cholesterol levels following bariatric surgery [26]. This discrepancy may be attributed to differences in preoperative LDLc levels, as suggested by previous research [25].

In this study, quality of life was assessed using the validated SF-36 questionnaire, which encompasses both physical and mental dimensions. Results showed a significant improvement in both the PCS and MCS at 6 months post-surgery, consistent with previous literature [27]. However, this improvement was not uniformly maintained. Thus, while the PCS remained relatively stable up to 24 months, the MCS experienced a significant decline during this period. These results suggest that the initial improvements in mental quality of life may not be sustained in the mid-term, in contrast to the stability observed in physical quality of life. Several factors may underlie these divergent trajectories. From a physiological standpoint, the maintenance of physical improvements may be largely explained by the sustained weight loss and reduction in obesity-related comorbidities typically observed after RYGB. Conversely, the decline in mental health scores could reflect the cumulative burden of nutritional restrictions, gastrointestinal side effects, or the development of micronutrient deficiencies, which can directly affect mood and cognition. From a psychosocial perspective, difficulties adapting to the new lifestyle, frustrations related to unmet expectations, the emergence of body dysmorphia, or a lack of emotional support from social or family environments. Longitudinal studies have shown that without complementary psychological interventions, a considerable proportion of patients may develop anxiety, depression, or eating disorders during the late postoperative phase [28,29]. Notably, physical role, bodily pain, and general health scores declined significantly at 24 months, with physical functioning being the only component that remained stable across all postoperative visits. This pattern suggests that although RYGB is effective at improving overall physical health in the short term, specific aspects such as pain management, role limitations, and mid-term musculoskeletal health require ongoing monitoring and supportive care. These findings highlight the need for a multidisciplinary follow-up approach—integrating nutritional counseling, physical activity programs, and psychological support—to maximize and sustain both the physical and mental health benefits of bariatric surgery.

From a nutritional perspective, longitudinal follow-up demonstrated a substantial and sustained reduction in total energy intake, as well as in carbohydrate and fat consumption. This reduction was most pronounced during the first postoperative year and stabilized during the second year. Conversely, protein intake showed an increasing trend between 12 and 24 months post-surgery, possibly reflecting greater awareness of the need to preserve lean mass or improved adherence to dietary recommendations. This contrasts with other studies, such as the work of Barstad et al. [30], where protein intake declined over time. In that study, a greater reduction in total energy intake was observed one year after bariatric surgery, mainly due to decreased intake of carbohydrates, fats, and protein, unlike in the present study, where protein intake levels were better maintained. Additionally, reductions in sodium, phosphorus, and potassium intake were observed, consistent with findings from other studies [30]. However, by two years post-surgery, these values tended to increase again, suggesting a gradual dietary normalization or a decline in adherence to nutritional recommendations. Notably, the decline in B vitamins (riboflavin, thiamine, B6) and minerals such as iron, zinc, calcium, iodine, and selenium observed here has not been reported in previous studies [30]. These findings highlight the need for continuous dietary monitoring after RYGB to prevent nutritional deficiencies and maintain balanced nutrition, since the lack of these micronutrients could slow weight loss, and in turn, promote the appearance of neuropsychological disorders.

Another relevant finding was the strong correlation between the consumption of certain saturated fatty acids, such as palmitic and stearic acid, and BMI, HOMA-IR, and insulin levels. This suggests that not only the quantity but also the quality of the diet plays a key role in the postoperative metabolic profile. In this regard, dietary patterns rich in saturated fats may attenuate some of the metabolic benefits derived from surgery, highlighting the importance of incorporating nutritional education strategies focused on qualitative food selection. However, the specific dietary patterns associated with sustained weight loss or weight regain remain unclear. Therefore, individualized recommendations regarding macronutrient intake and overall diet composition should be provided after bariatric surgery [31].

One limitation of this study is the use of bioelectrical impedance analysis (BIA), an indirect method for assessing body composition. Although BIA may underestimate body fat percentage, it is widely considered a suitable tool for monitoring longitudinal changes and evaluating group-level trends [32]. Another limitation is the loss of a considerable number of patients during follow-up, as many individuals typically discontinue clinical follow-up beyond two years after bariatric surgery. Nevertheless, enough patients were retained to enable statistically meaningful and robust comparisons across the assessed time points. A strength of our study is the extended follow-up of 24 months, providing insights into mid-term outcomes, whereas most previous studies report data only up to 12 months. This longer follow-up allows for a more comprehensive evaluation of changes in both clinical outcomes and quality of life after bariatric surgery.

Taken together, patients with severe obesity undergoing bariatric surgery experience significant weight loss, including reductions in both fat and lean mass compartments, along with improvements in glycemic, lipid, hepatic, and inflammatory parameters. These benefits are sustained up to 24 months following the intervention. While physical quality of life remains stable over time, initial improvements in mental quality of life may not be maintained in the mid-term.

## 5. Conclusions

In conclusion, RYGB resulted in substantial and sustained improvements in anthropometric, clinical, biochemical, nutritional, and the physical component of quality-of-life parameters at 24 months. These findings reinforce the effectiveness of bariatric surgery as a mid-term therapeutic intervention for patients with severe obesity. Nevertheless, our results also highlight that surgery should not be considered in isolation, as mental health may decline significantly at 24 months despite a remarkable increase at 6–12 months post- surgery. Long-term success is maximized when the procedure is integrated into a comprehensive care model that includes not only continuous nutritional guidance but also psychological support. Ongoing support from multidisciplinary teams, including endocrinologists, nutritionists, and psychologists, is essential to maintain initial benefits and prevent the risk of relapse.

## Figures and Tables

**Table 1:** Anthropometric changes, body composition, and metabolic parameters at 6, 12, and 24 months after bariatric surgery.

Variable	Preoperative (n = 95)	6 Months Post-Surgery (n = 95)	12 Months Post-Surgery (n = 95)	24 Months Post-Surgery (n = 95)	*p*-Value (Pre vs. 6 m)	*p*-Value (Pre vs. 12 m)	*p*-Value (Pre vs. 24 m)
Body weight, kg	116.1 (18)	85.2 (14.9)	77 (15.2)	77.3 (16.6)	<0.001	<0.001	<0.001
BMI, kg/m^2^	42.9 (5.3)	31.5 (4.7)	28.4 (4.9)	28.6 (5.4)	<0.001	<0.001	<0.001
Total weight lost, %	-	26.5 (5.9)	33.4 (8.4)	33.3 (9.6)	-	-	-
Excess weight lost, %	-	62.5 (19.3)	78.4 (25.2)	78.2 (26.9)	-	-	-
Waist, cm	124.4 (12.4)	100.4 (11.4)	92.9 (13.4)	94.1 (13.5)	<0.001	<0.001	<0.001
Hips, cm	136.5 (14)	114.9 (10.6)	108.1 (11.5)	108.1 (12.1)	<0.001	<0.001	<0.001
Body fat percentage, %	44.1 (5.5)	34.1 (8.4)	29.1 (8.8)	29.8 (8.7)	<0.001	<0.001	<0.001
Fat mass, kg	50.8 (11.5)	30.4 (11.0)	23.6 (11.9)	24.3 (11.2)	<0.001	<0.001	<0.001
Fat-free mass, kg	64.6 (10.8)	55.6 (11.2)	53.9 (9.4)	53.2 (9.5)	<0.001	<0.001	<0.001
Total body water percentage, %	40.2 (4.7)	44.1 (7.0)	45.9 (6.8)	45.4 (6.6)	<0.001	<0.001	<0.001
Muscle mass, kg	61.5 (10.4)	52.9 (9.7)	51.2 (8.9)	51.1 (9.3)	<0.001	<0.001	<0.001
Basal metabolic rate, kcal	2003 (362)	1670.6 (298.3)	1589.3 (326.9)	1582.8 (335)	<0.001	<0.001	<0.001
Bone mass, kg	3.2 (0.5)	2.8 (0.5)	2.7 (0.4)	2.7 (0.5)	<0.001	<0.001	<0.001
Systolic blood pressure, mm Hg	138.9 (16.7)	126.4 (17.8)	123.5 (18.8)	124.1 (19.4)	<0.001	<0.001	<0.001
Diastolic blood pressure, mm Hg	84.2 (10.1)	75.9 (10.3)	73.3 (11.3)	76.1 (12.2)	<0.001	<0.001	<0.001
Arm circumference, cm	41.2 (4.7)	33.9 (4.5)	32 (4.6)	31.4 (4.5)	<0.001	<0.001	<0.001
Calf circumference, cm	46.1 (4.6)	40.9 (3.7)	39.6 (7.1)	38.5 (4)	<0.001	0.015	<0.001

Data are presented as mean (±standard deviation). Differences between study time points were considered significant at *p* < 0.05.

**Table 2:** Changes in biochemical profile at 6, 12, and 24 months after bariatric surgery.

Variable	Preoperative (n = 95)	6 Months Post-Surgery (n = 95)	12 Months Post-Surgery (n = 95)	24 Months Post-Surgery (n = 95)	*p*-Value (Pre vs. 6 m)	*p*-Value (Pre vs. 12 m)	*p*-Value (Pre vs. 24 m)
HbA1c, %	5.9 (1.1)	5.5 (0.8)	5.6 (0.9)	5.6 (1.0)	<0.001	<0.001	<0.001
Insulin, µUI/mL	15.5 (10.4)	9.3 (4.7)	8.1 (5.3)	7.9 (4.9)	<0.001	<0.001	<0.001
*C*-peptide, ng/mL	3.2 (1.3)	2.3 (0.9)	2.2 (1.1)	2.0 (0.8)	<0.001	<0.001	<0.001
HOMA-IR	3.9 (2.9)	2.1 (1.3)	1.8 (1.3)	1.9 (2.5)	<0.001	<0.001	<0.001
Total cholesterol, mg/dL	163.0 (31.6)	159.4 (29.5)	158.5 (28.7)	165.2 (30.1)	0.164	0.120	0.335
Triglycerides, mg/dL	182.6 (98.9)	98.2 (41.4)	87.5 (43.8)	84.8 (40.0)	<0.001	<0.001	<0.001
HDL-chol, mg/dL	43.5 (10.5)	51.1 (9.0)	58.3 (11.1)	62.5 (11.4)	<0.001	<0.001	<0.001
LDL-chol, mg/dL	86.6 (29.1)	89.2 (25.4)	83.1 (23.3)	86.4 (26.1)	0.228	0.134	0.405
AST, U/L	20.9 (8.9)	20.7 (10.3)	21.4 (11.5)	20.5 (5.7)	0.431	0.383	0.320
ALT, U/L	22.9 (12.3)	21.9 (16.0)	25.4 (22.3)	20.5 (9.0)	0.296	0.221	0.049
GGT, U/L	24.5 (23.8)	15.6 (12.1)	16.2 (12.6)	15.5 (12.5)	<0.001	<0.001	<0.001
Ferritin, mg/mL	119.1 (147.2)	112.9 (139.8)	102.4 (113.3)	68.2 (97.7)	0.234	0.028	<0.001
Transferrin, mg/dL	257.5 (51.1)	256.9 (49.0)	266.8 (47.2)	286.4 (45.7)	0.450	0.059	<0.001
TSAT, %	24.7 (10.7)	20.4 (8.4)	22.9 (13.1)	21.8 (9.2)	<0.001	0.161	0.056
Albumin, g/dL	4.1 (0.4)	4.4 (0.2)	4.5 (0.3)	4.4 (0.3)	<0.001	<0.001	<0.001
Total protein, g/dL	6.5 (0.6)	6.7 (0.4)	6.7 (0.4)	6.7 (0.4)	<0.001	0.006	0.001
Folate, ng/mL	8.8 (4.9)	11.8 (5.0)	11.4 (4.9)	11.9 (5.4)	<0.001	<0.001	<0.001
Vitamin B12, pg/mL	411.0 (150.5)	403.3 (130.1)	403.9 (155.0)	448.3 (117.2)	0.263	0.335	0.009
Uric acid, mg/dL	5.5 (1.4)	4.5 (1.1)	3.9 (1.2)	3.9 (1.2)	<0.001	<0.001	<0.001
Creatinine, mg/dL	0.7 (0.2)	0.7 (0.2)	0.7 (0.2)	0.7 (0.2)	0.067	0.170	0.225
CRP, mg/dL	0.7 (0.6)	0.3 (0.3)	0.2 (0.3)	0.1 (0.4)	<0.001	<0.001	<0.001

Data are presented as mean (±standard deviation). Differences between study time points were considered significant at *p* < 0.05. Abbreviations: HbA1c, glycated hemoglobin; HDL-C, high-density lipoprotein cholesterol; TSAT, transferrin saturation index; LDL-C, low-density lipoprotein cholesterol; CRP: *C*-reactive protein.

**Table 3:** Changes in dietary intake of micronutrients and alcohol in patients undergoing bariatric surgery.

Variable	Preoperative (n = 95)	12 Months Post-Surgery (n = 95)	24 Months Post-Surgery (n = 95)	*p*-Value (Pre vs. 12 m)	*p*-Value (Pre vs. 24 m)	*p*-Value (12 m vs. 24 m)
Alcohol, g/day	4.5 (8.9)	1.7 (5.1)	2.6 (6.4)	<0.001	0.007	0.016
Cholesterol, mg/day	788.4 (644.1)	520.5 (313.1)	639.8 (433.4)	<0.001	0.025	0.003
Niacin, mg/day	127.7 (88.9)	127.2 (73.5)	157.0 (98.6)	0.482	0.008	0.004
Folate, µg/day	645.1 (365.4)	716.5 (322.3)	734.3 (491.7)	0.053	0.062	0.356
Riboflavin, mg/day	3.8 (2.1)	3.1 (1.3)	3.4 (1.5)	0.004	0.041	0.075
Thiamine, mg/day	2.9 (1.7)	2.2 (0.9)	2.4 (1.2)	<0.001	0.004	0.066
Vitamin A (retinol equivalent), µg/day	1987.0 (1409.5)	1844.5 (1314.1)	2021.9 (1460.4)	0.235	0.426	0.173
Vitamin B12, µg/day	16.9 (11.7)	17.4 (10.9)	21.1 (13.7)	0.396	0.008	0.014
Vitamin B6, mg/day	5.4 (2.8)	4.7 (1.8)	5.3 (2.5)	0.022	0.450	0.012
Vitamin C, mg/day	380.9 (254.7)	502.1 (296.6)	494.4 (400.4)	<0.001	0.006	0.424
Vitamin D, µg/day	9.8 (8.6)	10.2 (7.7)	13.2 (10.4)	0.357	0.004	0.006
Vitamin E, mg/day	36.9 (22.8)	36.3 (22.2)	37.6 (21.9)	0.422	0.421	0.307
Phosphorus, mg/day	3118.6 (1677.6)	2486.8 (1010.2)	2751.4 (1282.4)	<0.001	0.025	0.033
Iron, mg/day	33.1 (19.7)	27.8 (10.0)	30.6 (16.6)	0.007	0.150	0.058
Iodine, µg/day	210.9 (118.6)	179.8 (67.2)	203.0 (103.1)	0.006	0.275	0.012
Magnesium, mg/day	713.8 (414.4)	665.5 (323.5)	705.2 (353.1)	0.162	0.432	0.157
Potassium, mg/day	7264.4 (3896.0)	6842.5 (2513.4)	7163.8 (3626.3)	0.156	0.413	0.191
Selenium, mg/day	249.2 (149.6)	197.4 (84.7)	223.5 (130.9)	0.001	0.073	0.033
Sodium, mg/day	4846.3 (3319.8)	2489.0 (1290.6)	2975.1 (1845.1)	<0.001	<0.001	0.010
Zinc, mg/day	19.8 (10.6)	15.5 (6.5)	16.9 (8.0)	<0.001	0.009	0.049
Calcium, mg/day	2006.3 (1211.9)	1552.7 (627.8)	1644.5 (779.5)	<0.001	0.003	0.137

Data are presented as mean (±standard deviation). Differences between study time points were considered significant at *p* < 0.05.

**Table 4:** Changes in fatty acid consumption before and after bariatric surgery.

Variable	Preoperative (n = 95)	12 Months Post-Surgery (n = 95)	24 Months Post-Surgery (n = 95)	*p*-Value (Pre vs. 12 m)	*p*-Value (Pre vs. 24 m)	*p*-Value (12 m vs. 24 m)
Monounsaturated fatty acids, g/day	85.7 (57.8)	55.5 (35.0)	56.6 (34.7)	<0.001	<0.001	0.390
Polyunsaturated fatty acids, g/day	37.2 (27.5)	25.3 (22.4)	26.8 (17.2)	<0.001	<0.001	0.255
Saturated fatty acids, g/day	61.3 (45.6)	29.3 (14.6)	33.4 (18.1)	<0.001	<0.001	0.030
Lauric acid (12:0), g/day	2.2 (3.0)	0.4 (0.6)	0.4 (0.6)	<0.001	<0.001	0.264
Myristic acid (14:0), g/day	2.1 (2.4)	0.5 (0.6)	0.7 (0.6)	<0.001	<0.001	0.041
Palmitic acid (16:0), g/day	20.4 (17.7)	8.7 (6.0)	9.2 (6.4)	<0.001	<0.001	0.257
Stearic acid (18:0), g/day	7.5 (6.8)	2.8 (2.2)	3.1 (2.3)	<0.001	<0.001	0.138
Oleic acid (18:1 *n*-9), g/day	56.7 (43.1)	39.0 (31.5)	36.2 (29.1)	<0.001	<0.001	0.201
Arachidonic acid (20:4 *n*-6), g/day	0.13 (0.12)	0.09 (0.10)	0.11 (0.10)	0.013	0.083	0.129
Eicosapentaenoic acid (20:5), g/day	0.29 (0.27)	0.39 (0.26)	0.44 (0.38)	0.003	<0.001	0.103
Docosahexaenoic acid (22:6 *n*-3), g/day	0.55 (0.51)	0.76 (0.45)	0.87 (0.68)	0.001	<0.001	0.068

Data are presented as mean (±standard deviation). Differences between study time points were considered significant at *p* < 0.05.

## Data Availability

All data generated or analyzed during this study are included in this article. Further enquiries can be directed to the corresponding author.

## Abbreviations

The following abbreviations are used in this manuscript:

RYGB	Roux-en Y-gastric bypass
BMI	Body Mass Index
CVD	Cardiovascular disease
TWL	Total weight loss
EWL	Excess weight loss
BI	Broca index
6MWT	Six-minute walk test
HDLc	High-density lipoprotein cholesterol
LDLc	Low-density lipoprotein cholesterol
TG	Triglycerides
ALT	Alanine aminotransferase
AST	Aspartate aminotransferase
GGT	Gamm-glutamyl transferase
Apo-Ai	Apolipoprotein A-I
Apo-B	Apolipoprotein B
HbA1c	Glycated hemoglobin
FFQ	Frequency questionnaire
PCS	Physical component summary
MCS	Mental component summary
ANOVA	Analysis of variance
EPA	Eicosapentaenoic acid
BIA	Bioelectrical impedance analysis

## References

[1] de Andrade Mesquita L., Wayerbacher L.F., Schwartsmann G., Gerchman F. (2023). Obesity, diabetes, and cancer: Epidemiology, pathophysiology, and potential interventions. Arch. Endocrinol. Metab..

[2] Gutiérrez-González E., García-Solano M., Rollán-Gordo A., Peña-Rey I. (2023). Estudio ENE-COVID: Situación Ponderal de la Población Adulta.

[3] Lecube A., Azriel S., Barreiro E., Blay G. (2024). Guía Española GIRO: Guía Española del Manejo Integral y Multidisciplinar de la Obesidad en Personas Adultas.

[4] Głuszek S., Bociek A., Suliga E., Matykiewicz J., Kołomańska M., Bryk P., Znamirowski P., Nawacki Ł., Głuszek-Osuch M., Wawrzycka I. (2020). The effect of bariatric surgery on weight loss and metabolic changes in adults with obesity. Int. J. Environ. Res. Public Health.

[5] Haddad A., Suter M., Greve J.W., Shikora S., Prager G., Dayyeh B.A., Galvao M., Grothe K., Herrera M., Kow L. (2024). Therapeutic Options for Recurrence of Weight and Obesity Related Complications After Metabolic and Bariatric Surgery: An IFSO Position Statement. Obes. Surg..

[6] Salminen P., Kow L., Aminian A., Kaplan L.M., Nimeri A., Prager G., Behrens E., White K.P., Shikora S., Dayyeh B.K.A. (2024). IFSO Consensus on Definitions and Clinical Practice Guidelines for Obesity Management—An International Delphi Study. Obes. Surg..

[7] Noria S.F., Shelby R.D., Atkins K.D., Nguyen N.T., Gadde K.M. (2023). Weight Regain After Bariatric Surgery: Scope of the Problem, Causes, Prevention, and Treatment. Curr. Diab. Rep..

[8] Nymo S., Lundanes J., Aukan M., Sandvik J., Johnsen G., Græslie H., Larsson I., Martins C. (2022). Diet and physical activity are associated with suboptimal weight loss and weight regain 10–15 years after Roux-en-Y gastric bypass: A cross-sectional study. Obes. Res. Clin. Pract..

[9] Nymo S., Børresen Skjølsvold O., Aukan M., Finlayson G., Græslie H., Mårvik R., Kulseng B., Sandvik J., Martins C. (2022). Suboptimal Weight Loss 13 Years After Roux-en-Y Gastric Bypass: Is Hedonic Hunger, Eating Behaviour and Food Reward to Blame?. Obes. Surg..

[10] Major P., Matłok M., Pędziwiatr M., Migaczewski M., Budzyński P., Stanek M., Kisielewski M., Natkaniec M., Budzyński A. (2015). Quality of Life After Bariatric Surgery. Obes. Surg..

[11] Eiser C., Jenney M. (2007). Measuring quality of life. Arch. Dis. Child..

[12] Urbach D.R. (2005). Measuring quality of life after surgery. Surg. Innov..

[13] Saarinen I., Strandberg M., Hurme S., Helmiö M., Grönroos S., Juuti A., Juusela R., Nuutila P., Salminen P. (2025). Nutritional deficiencies after sleeve gastrectomy and Roux-en-Y gastric bypass at 10 years: Secondary analysis of the SLEEVEPASS randomized clinical trial. Br. J. Surg..

[14] Mechanick J.I., Apovian C., Brethauer S., Garvey W.T., Joffe A.M., Kim J., Kushner R.F., Lindquist R., Pessah-Pollack R., Seger J. (2020). Clinical practice guidelines for the perioperative nutrition, metabolic, and nonsurgical support of patients undergoing bariatric procedures—2019 update: Cosponsored by American Association of Clinical Endocrinologists/American College of Endocrinology. Surg. Obes. Relat. Dis..

[15] Tangjittrong S., Udomsawaengsup S., Boonchaya-anant P. (2023). Comparison of Body Composition Variables between Post-Bariatric Surgery Patients and Non-Operative Controls. Clin. Med. Insights Endocrinol. Diabetes.

[16] Hojaji E., Veysi Z., Fe’li S.N., Shalbaf N., Arian M., Clark C.C.T., Dorosty Motlagh A.R. (2025). Evaluation of nutritional, anthropometric, and psychological outcomes in different metabolic and bariatric surgery techniques: A follow up study. BMC Surg..

[17] Issues S., Test M.W., Equipment R., Preparation P. (2002). American Thoracic Society ATS Statement: Guidelines for the Six-Minute Walk Test. Am. J. Respir. Crit. Care Med..

[18] Martin-moreno J.M., Boyle P., Gorgojo L., Maisonneuve P., Fernandez-rodriguez J.C., Salvini S., Willett W.C. (1993). Development and validation of a food frequency questionnaire in Spain. Int. J. Epidemiol..

[19] Mataix-Solera J., Manas M., Llopis J., de Victoria E.M. (1998). Tabla de Composición de Alimentos Españoles.

[20] Coulman K.D., Abdelrahman T., Owen-Smith A., Andrews R.C., Welbourn R., Blazeby J.M. (2013). Patient-reported outcomes in bariatric surgery: A systematic review of standards of reporting. Obes. Rev..

[21] Benaiges D., Parri A., Subirana I., Pedro-Botet J., Villatoro M., Ramon J.M., Climent E., Flores Le Roux J.A., Goday A. (2020). Most of qualitative dietary changes observed one year post-bariatric surgery can be achieved with a preoperative dietary intervention. Endocrinol. Diabetes Nutr..

[22] Strain G.W., Ebel F., Honohan J., Gagner M., Dakin G.F., Pomp A., Gallagher D. (2017). Fat-free mass is not lower 24 months postbariatric surgery than nonoperated matched controls. Surg. Obes. Relat. Dis..

[23] Jassil F.C., Papageorgiou M., Mackay E., Carnemolla A., Kingett H., Doyle J., Kirk A., Lewis N., Montagut G., Marvasti P. (2025). One Year Changes in Body Composition and Musculoskeletal Health Following Metabolic/Bariatric Surgery. J. Clin. Endocrinol. Metab..

[24] de Souza S.A.F., Faintuch J., Fabris S.M., Nampo F.K., Luz C., Fabio T.L., Sitta I.S., de Batista Fonseca I.C. (2009). Six-minute walk test: Functional capacity of severely obese before and after bariatric surgery. Surg. Obes. Relat. Dis..

[25] Cunha J.B., Fialho M.C.M.P., Arruda S.L.M., Nóbrega O.T., Camargos E.F. (2020). Clinical and Metabolic Improvement after Bariatric Surgery in Older Adults: A 6-Year Follow-Up. J. Nutr. Heal. Aging.

[26] Nascimento I., Padilha B., Araujo M.L., Silva P.C., Noronha G.A., Cabral P.C., Ferraz A.A.B. (2022). Vitamin Levels And Lipid Profile In Patients Undergoing Surgery. Arq. Bras. Cir. Dig..

[27] Sellberg F., Possmark S., Willmer M., Tynelius P., Berglind D. (2019). One-year follow-up of a dissonance-based intervention on quality of life, wellbeing, and physical activity after Roux-en-Y gastric bypass surgery: A randomized controlled trial. Surg. Obes. Relat. Dis..

[28] De Zwaan M., Enderle J., Wagner S., Mühlhans B., Ditzen B., Gefeller O., Mitchell J.E., Müller A. (2011). Anxiety and depression in bariatric surgery patients: A prospective, follow-up study using structured clinical interviews. J. Affect. Disord..

[29] Kikuchi J.L.D., de Lima Carvalhal M.M., da Silva Costa A.P., Vasconcelos J.A.S.B., Paracampo C.C.P., Gomes D.L. (2022). Correlation between Anxiety Symptoms and Perception of Quality of Life in Women with More Than 24 Months after Undergoing Bariatric Surgery. Int. J. Environ. Res. Public Health.

[30] Barstad L.H., Johnson L.K., Borgeraas H., Hofsø D., Svanevik M., Småstuen M.C., Hertel J.K., Hjelmesæth J. (2023). Changes in dietary intake, food tolerance, hedonic hunger, binge eating problems, and gastrointestinal symptoms after sleeve gastrectomy compared with after gastric bypass; 1-year results from the Oseberg study—A randomized controlled trial. Am. J. Clin. Nutr..

[31] Cheung H.C., Strodl E., Musial J., MacLaughlin H.L., Byrnes A., Lewis C.A., Ross L.J. (2023). Associations between diet composition, dietary pattern, and weight outcomes after bariatric surgery: A systematic review. Int. J. Obes..

[32] Widen E.M., Strain G., King W.C., Yu W., Lin S., Goodpaster B., Thornton J., Courcoulas A., Pomp A., Gallagher D. (2014). Validity of Bioelectrical Impedance Analysis for Measuring Changes in Body Water and Percent Fat after Bariatric Surgery. Obes. Surg..

